# How bumblebees use lateral and ventral optic flow cues for position control in environments of different proximity

**DOI:** 10.1007/s00359-017-1173-9

**Published:** 2017-04-20

**Authors:** Nellie Linander, Emily Baird, Marie Dacke

**Affiliations:** Department of Biology, Lund Vision Group, Lund University, Lund, Sweden

**Keywords:** Flight control, Optic flow, *Bombus terrestris*, Position control, Centring

## Abstract

Flying insects frequently navigate through environments of different complexity. In this study, buff-tailed bumblebees (*Bombus terrestris* L.) were trained to fly along tunnels of different widths, from 60 to 240 cm. In tunnel widths of 60 and 120 cm, bumblebees control their lateral position by balancing the magnitude of translational optic flow experienced in the lateral visual field of each eye. In wider tunnels, bumblebees use translational optic flow cues in the ventral visual field to control their lateral position and to steer along straight tracks. Our results also suggest that bumblebees prefer to fly over surfaces that provide strong ventral optic flow cues, rather than over featureless ones. Together, these strategies allow bumblebees to minimize the risk of collision and to maintain relatively straight flight paths in a broad range of environments.

## Introduction

Flying insects, such as bees, frequently navigate through environments of different complexity, from open fields to cluttered bushes. They rely heavily on the optic flow information (the pattern of apparent image motion generated across the retina as an animal moves through its world) generated by translational motion to adjust their speed, position, and height in response to changes in the proximity of nearby obstacles (e.g., Baird et al. 2010; Dyhr and Higgins 2010; Portelli et al. 2010, 2011; Linander et al. 2015, 2016, for reviews see Srinivasan et al. 1996; Srinivasan and Zhang 2000; Srinivasan 2011). Optic flow resulting from translational motion is important for this task as it provides information about relative distance to nearby surfaces, forward speed, and the spatial layout of the environment (Gibson 1950, 1979; Koenderink 1986; Lappe 2000; Collett 2002).

Studies investigating flight control in free flying insects have primarily been conducted in flight tunnels that are 40 cm or less in width. When flying along these narrow corridors, honeybees (e.g., Kirchner and Srinivasan 1989; Srinivasan et al. 1991) and bumblebees (Dyhr and Higgins 2010; Linander et al. 2015) appear to control their lateral position by balancing the magnitude of lateral optic flow experienced in each eye, which enables them to keep an equal distance from each wall. In nature, however, bees very often encounter more open environments. Honeybees will fly along the midline of a 95 cm-wide tunnel if both the feeder and the entrance are centred, but if the feeder and the entrance are placed on the same side of the tunnel, they will adopt a wall-following behaviour, suggesting that they do not necessarily balance the magnitude of lateral optic flow in each eye when flying in more open spaces (Serres et al. 2008). A more recent study investigating position control in bumblebees flying to a hidden feeder, however, found that the flights remain centred about the midline, even when the distance between the walls reaches 120 cm (Linander et al. 2016). This study also showed that, in tunnels wider than 120 cm, lateral position becomes increasingly variable and the bees are less likely to maintain an equal distance to the two walls. The strong correlation between ground speed and height in these wider tunnels suggests that, as the distance between the walls becomes greater, bumblebees rely increasingly on optic flow information from the ventral visual field to control their speed. Although this study showed the importance of optic flow information in the ventral visual field for speed control in open environments, what remains unclear is whether ventral optic flow cues can also be used for lateral position control in open environments. If so, how effective are ventral optic flow cues for maintaining a straight flight path and are these cues alone sufficient for controlling position?

The aim of the present study is to answer these questions by investigating the role of ventral optic flow cues in the control of lateral position in environments of different complexity and proximity. We do this by varying the lateral and ventral optic flow information available to buff-tailed bumblebees (*Bombus terrestris* L.) flying along experimental tunnels of different widths (from 60 to 240 cm) and analysing the effect on lateral position.

## Materials and methods

### Experimental setup

The experiments were conducted in a white tent (6 m long, 3 m wide, and 2 m high) placed inside a greenhouse in Lund, Sweden (May to August 2014 and 2016). The light intensity and temperature (2273 ± 1396 lx, 22 ± 3 °C, mean ± SD) in the tent varied somewhat according to natural fluctuations in the weather. A bumblebee hive (*B. terrestris* L. from Koppert UK) was placed at the centre of the entrance to a 5 m-long flight tunnel with walls that could be set to different widths and heights (60, 120, 180, and 240 cm). The height of the walls was held equal to the distance between them, except for the 240 cm-wide tunnel that had a wall height of 180 cm due to space constraints. A total of six hives were used throughout the experiment. The bees were trained to fly along the tunnel to a feeder that was hidden from view in a white box. The feeder consisted of two channels (one for sugar water and one for pollen) that ran across the width of the tunnel. The top of the tunnel was covered with black insect netting and the only visible structures above the tunnel were three metal bars supporting the roof of the tent, and a camera (Mikrotron MotionBLITZ EoSens, Unterschleißheim, Germany) that was mounted above the centre of the tunnel to record flights towards the feeder at 80 Hz.

**Fig. 1:**
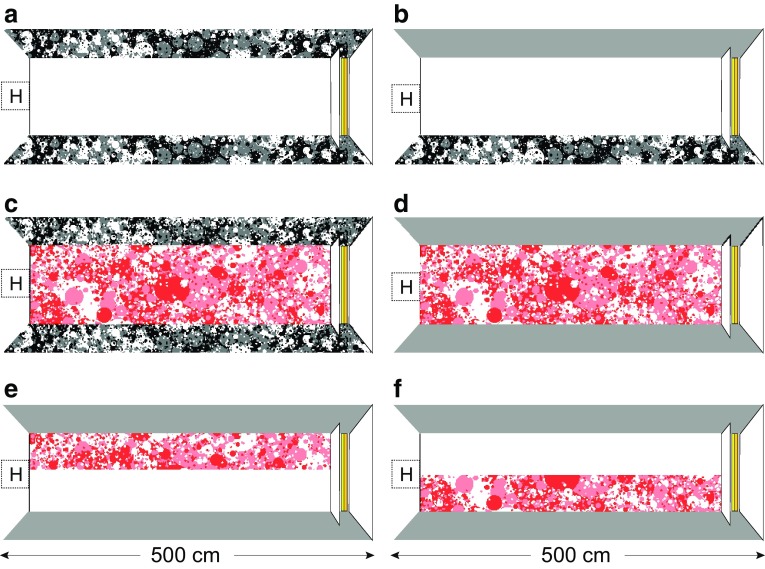
Experimental setup and the pattern combinations used in different conditions. The bumblebee hives (H) were placed at the middle of the entrance to a 5 m-long flight tunnel with flexible height and width (60, 120, 180, and 240 cm). The feeder channels (marked in *yellow* for nectar and *orange* for pollen) were hidden in a white box at the end of the flight tunnel. A high-speed camera recorded bees flying over the central (150 cm) section of the tunnel. The floor of the tunnel was either *white* (**a**, **b**), lined with a *red* *dead leaves pattern* that covered either the entire width (**c**, **d**) or half the width (**e**, **f**) of the tunnel floor. The walls were either *grey *(**b**, **d**–**f**) or lined with a *black* *dead leaves pattern* (**a**, **c**). Conditions with a completely patterned floor and patterned walls (**c**) are re-analysed from Linander et al. (2016)

Depending on the experimental condition, the walls of the flight tunnel were lined with a uniformly grey pattern or a black-and-white “dead leaves” pattern that was specifically designed to ensure that optic flow cues were available to the bees at all distances from the tunnel walls (for technical specifications, see Lee et al. 2001). The walls of the flight tunnel were lined with a black-and-white pattern to enable a direct comparison with the previous studies that used the same arrangement (e.g., Linander et al. 2015, 2016). To minimize lateral optic flow input, the walls displayed a uniform grey pattern. The floor of the flight tunnel was white or lined with a red-and-white dead leaves pattern that facilitated the tracking of the bees in the recorded sequences while still maintaining a high pattern contrast. We lined the floor with white to enable comparison with earlier studies (e.g., Linander et al. 2015, 2016). Figure 1 illustrates the different experimental conditions for one tunnel width.

### Analysis of flight trajectories

Flight trajectories were analysed over a distance of 150 cm (75 cm before and 75 cm after the midsection of the tunnel). In each video frame, the centre of mass of the bumblebee was determined (in *x*- and *y*-pixel coordinates) using an automated tracking program (Lindemann 2005). Data were converted from pixels to mm using a reference pattern placed at the estimated average flight height of 30 cm above the floor. To minimize calculation errors, bees that flew higher than 60 cm above the floor (determined by the number of pixels they occupied in the recorded sequences) were excluded from further analysis. Flights with bees flying in pairs or on a collision course with another bee were also excluded from the analysis. Due to the size of the tunnel and feeder, it was not possible to collect and individually mark the bees; thus, to minimize pseudo-replication, a large number of flights (80–100 flights) were analysed for each condition. As there were many different foragers flying to the feeder during the experiments, the likelihood of including many flights from the same individuals is minimal. In addition, the previous studies with marked bumblebees have shown that intra-individual variation between flights is not different from inter-individual variation (e.g., Dyhr and Higgins 2010).

Lateral position was calculated with respect to the midline (positive and negative values indicate flights to the left and right sides of the midline, respectively). The within-flight variation in lateral position (the spread of lateral positions within each flight trajectory) was calculated using the 25–75% interquartile range for each flight and was used as a measure for how well a bee could control its lateral position along the length of its trajectory. The tortuosity of each flight trajectory was calculated as the ratio of the total distance travelled to the straight-line distance between the first and last positions. A completely straight flight path will have a tortuosity value of 1, while more curved or variable paths will result in values greater than 1. The density of lateral positions for all flights within a condition was calculated by assigning the lateral position recorded in each video frame into grid-elements corresponding to 5% of the tunnel width. The relative frequency of data points in each grid element (that is, the relative frequency of flight passages) was averaged over the tunnel length to create a histogram of the spatial distribution of the flight trajectories across the width of the tunnel. The relative frequency of flight passages is the absolute number of flight passages normalized by the total number of flight passages.

Wilcoxon rank-sum tests were used to compare the data from two conditions. Kruskal–Wallis tests were used to compare the within-flight variation in lateral position between conditions. All significance levels were set to *p* < 0.05, and a Bonferroni correction was applied when multiple comparisons were made on the same data set.

To control for a possible side bias in the data, the lateral positions obtained from flights when only one of the walls (right or left) was lined with the dead leaves pattern (and the other grey) were inverted and compared. In all cases, the data were not significantly different, indicating that there was no side bias in the data set. To simplify the analysis, the data were pooled and presented as one condition with the grey pattern along the left side. Flight positions from the experimental conditions with a patterned floor and walls (Fig. 1c) were re-analysed from Linander et al. (2016).

## Results

### The role of lateral optic flow for position control in environments of different proximity

**Fig. 2:**
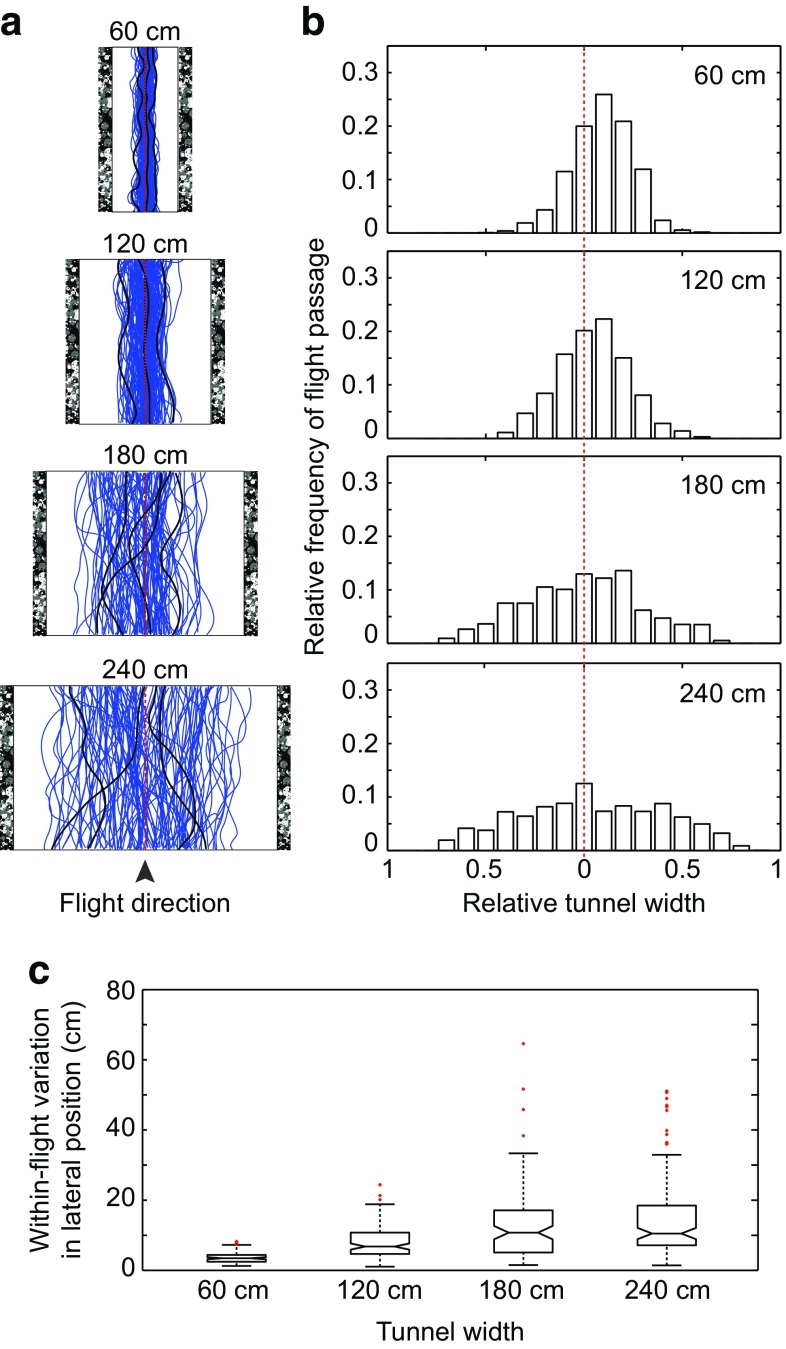
Effect of tunnel width on lateral position. Both tunnel walls were lined with a *dead leaves pattern* and the floor was *white*. **a** Raw flight trajectories of bees flying in the 60, 120, 180, and 240 cm-wide tunnels. Some individual examples are highlighted in *black*. **b** Relative frequency of flight passage in tunnels of different width. *Each bar* corresponds to a longitudinal strip that has a relative width of 5% of the tunnel diameter. The *red dotted line* represents the *midline* of the tunnel. **c** Within-flight variation (25–75% interquartile range) in lateral position. *Boxes* indicate the extent of the 25–75% interquartile range, the *horizontal line* indicates the median, *whiskers* indicate the full extent of the data, and *red crosses* represent outliers

In the first set of experiments, the walls of the tunnels were lined with the dead leaves pattern to provide strong optic flow cues in the lateral visual field, while the optic flow cues in the ventral visual field were minimized using a white floor (see Fig. 1a). For all tunnel widths (60, 120, 180, and 240), the flight trajectories meandered slightly (Fig. 2a), although the median lateral positions remained relatively close to the midline (Fig. 2b; Table 1). However, the within-flight variation in lateral position increased with tunnel width (Fig. 2c, Kruskal–Wallis $\chi_{3/433}^{2}$ = 147.6, *p* < 0.001). Moreover, in the two narrower tunnels (60 and 120 cm), the lateral positions were spread over no more than 50% of the tunnel width, whereas in the two wider tunnels (180 and 240 cm), the lateral positions were more dispersed, covering approximately 75% of the tunnel width (Fig. 2b). These results suggest that, in the absence of ventral optic flow, bumblebees’ ability to control their position becomes worse as the distance to nearby lateral surfaces generating optic flow increases.

**Table 1:** Details of lateral position and statistical analyses

Tunnel width (cm)	Condition			Lateral position (cm) median (interquartile range)	Wilcoxon statistics (a vs. b)	Kruskal–Wallis statistics (a vs. c)
	Floor		Walls			
60	a	White	Dead leaves	−1.3 (−3.6 to 1.4)	*Z* = −8.1 *p* < 0.001	$\chi_{1/203}^{2}$ = 18.5 *p* < 0.001
	b	White	Grey/dead leaves	3.6 (0.4 to 7.0)		
	c	Dead leaves	Dead leaves	1.4 (−0.6 to 3.0)		
120	a	White	Dead leaves	−1.4 (−6.4 to 5.9)	*Z* = −7.1 *p* < 0.001	$\chi_{1/223}^{2}$ = 34.6 *p* < 0.001
	b	White	Grey/dead leaves	8.5 (1.7 to 15.2)		
	c	Dead leaves	Dead leaves	−1.3 (−5.9 to 2.8)		
180	a	White	Dead leaves	1.2 (−12.1 to 23.1)	*Z* = −0.6 *p* = 0.56	$\chi_{1/207}^{2}$ = 25.5 *p* < 0.001
	b	White	Grey/dead leaves	5.7 (−9.5 to 22.9)		
	c	Dead leaves	Dead leaves	0.7 (−8.9 to 7.9)		
240	a	White	Dead leaves	6.1 (−29.8 to 28.6)	*Z* = −0.02 *p* = 0.98	$\chi_{1/241}^{2}$ = 9.04 *p* < 0.01
	b	White	Grey/dead leaves	1.3 (−25.6 to 27.5)		
	c	Dead leaves	Dead leaves	8.4 (−9.8 to 24.0)		

Data from the conditions with a patterned floor (c) are re-analysed from Linander et al. (2016)

**Fig. 3:**
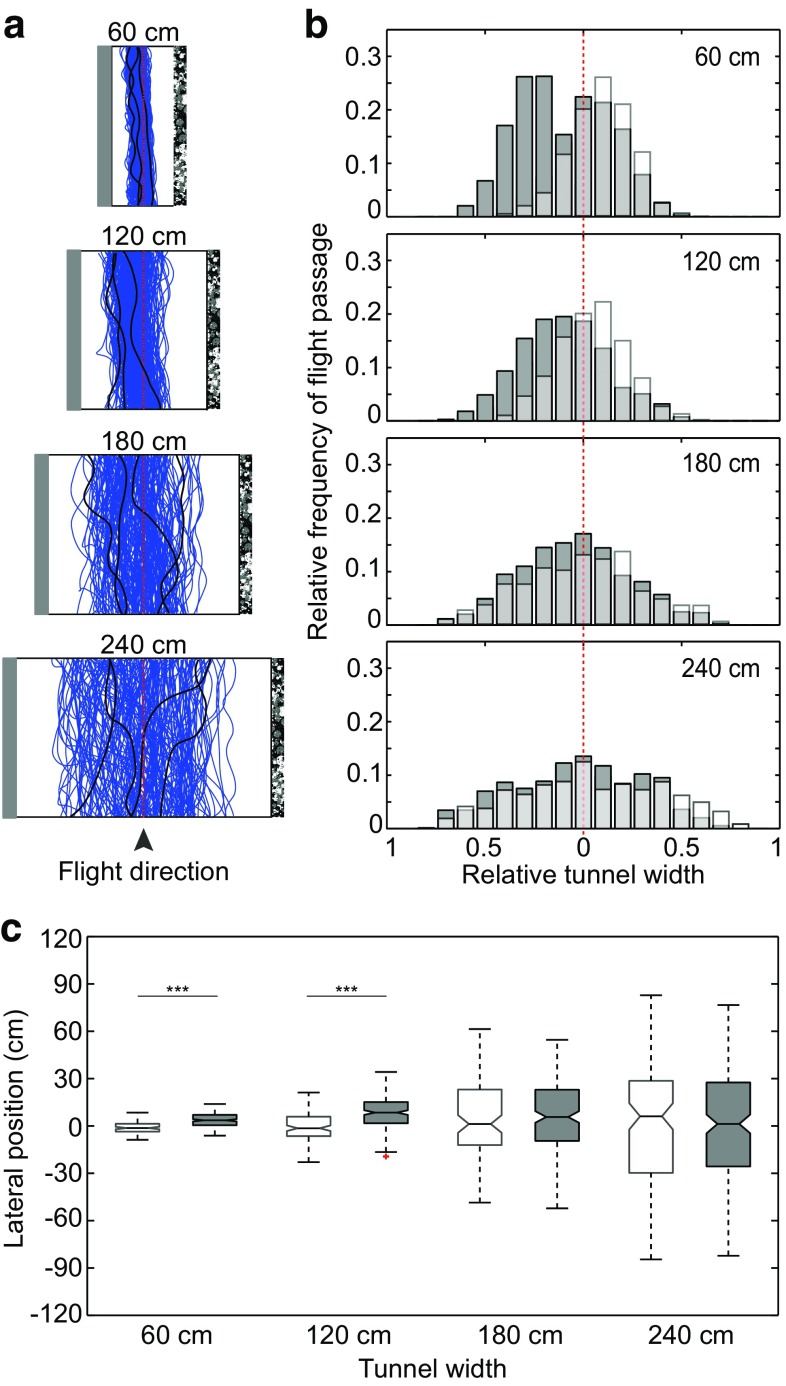
Effect of asymmetric optic flow input on lateral position. **a** Raw flight trajectories of bees flying in the 60, 120, 180, and 240 cm-wide tunnels when one wall was lined with a *dead leaves pattern* and the other wall was *grey*. The floor was *white*. Some individual examples are highlighted in *black*. **b** Relative frequency of flight passage in tunnels of different width. The *plot* contains data from two different conditions: *dark grey bars* represent the data from the condition in which the *right* tunnel wall was lined with *dead leaves pattern* and the *left* wall was lined with a homogenous *grey* pattern, *white transparent bars* represent the condition in which *both walls* were lined with *dead leaves pattern*. The *light grey areas* represent overlap between the two data sets. *Each bar* corresponds to a longitudinal strip that has a relative width of 5% of the tunnel diameter. The *red dotted line* represents the *midline* of the tunnel. **c** Median lateral position of bees flying in a tunnel lined with *dead leaves pattern* on *both walls* (*white boxes*) or in a tunnel lined with *dead leaves pattern* on the *right* wall and a homogenous *grey* pattern on the *left* wall (*grey boxes*). *Positive* and *negative values* indicate flights to the *left* and *right* sides of the *midline*, respectively. *Boxes* indicate the extent of the 25–75% interquartile range, the *horizontal line* indicates the median, *whiskers* indicate the full extent of the data, and *red crosses* represent outliers. *Asterisks* indicate the significance level (Wilcoxon rank-sum): ***p < 0.001

To further investigate the importance of lateral optic flow for the control of flight position in tunnels of different widths, we presented bees flying along the tunnels with asymmetric optic flow in the lateral visual field by replacing the pattern on one wall with a homogeneous grey pattern (see Fig. 1b). The previous work has shown that bumblebees control their position by balancing the magnitude of lateral optic flow experienced in each eye (Dyhr and Higgins 2010; Linander et al. 2015). If bumblebees rely on lateral optic flow cues to control their position in this experiment, then they will strive to balance the magnitude of optic flow by flying further away from the wall displaying the dead leaves pattern and closer to the grey wall. The results, indeed, show that, in the two narrow tunnels, the bees flew closer to the grey wall in comparison to the central flight paths recorded when both walls displayed the dead leaves pattern (Fig. 3, Wilcoxon rank-sum *p* < 0.001; for statistical details, see Table 1). In contrast, the flight trajectories in the two wider tunnels did not shift towards the grey wall (Fig. 3a, b), and the median lateral positions did not differ significantly from the positions recorded when both walls displayed the dead leaves pattern (Fig. 3c, Wilcoxon rank-sum *p* = 0.56 (180 cm) and *p* = 0.98 (240 cm); for statistical details, see Table 1). These results suggest that, in the absence of ventral optic flow cues, bumblebees do not control their position by balancing the relative magnitude of optic flow experienced in each lateral visual field when the distance to nearby lateral surfaces is large.

### The role of ventral optic flow for position control in environments of different proximity

**Fig. 4:**
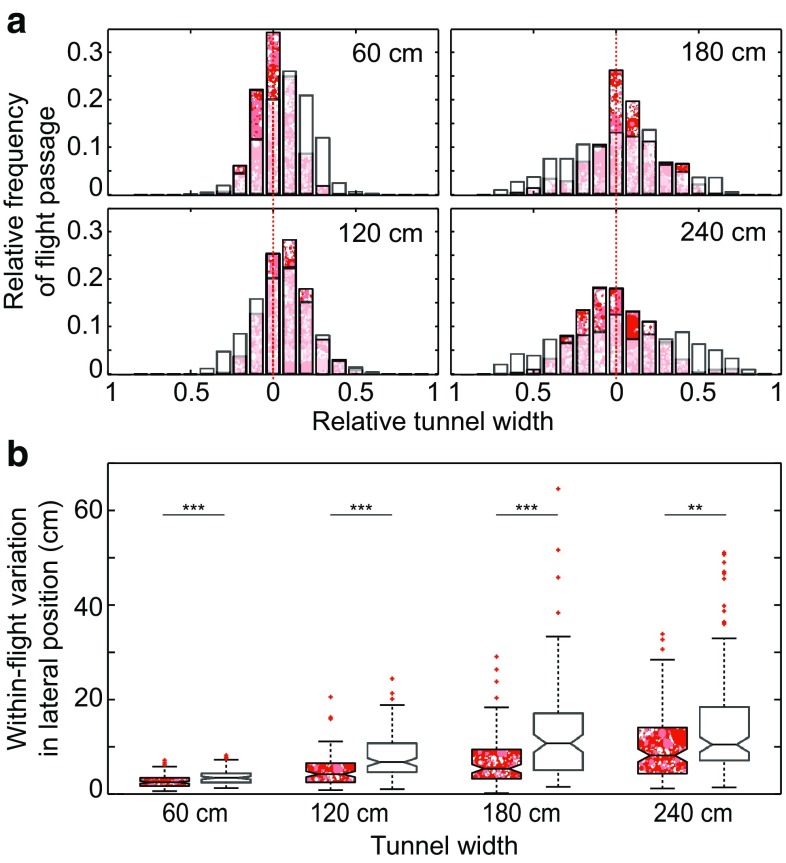
Effect of ventral optic flow on lateral position. The floor was either *white* or lined with a *red dead leaves pattern*. Both walls were lined with a *dead leaves pattern*. **a** Relative frequency of flight passage in tunnels of different width. The *plot* contains data from two different conditions: *dark red patterned bars* represent flights over a patterned floor (data from Linander et al. 2016), and *white transparent bars* represent flights over a *white* floor. The *light red patterned areas* represent overlap between the two data sets. *Each bar* corresponds to a longitudinal strip that has a relative width of 5% of the tunnel diameter. The *red dotted line* represents the *midline* of the tunnel. **b** Within-flight variation (interquartile range) in lateral position for bees flying in a tunnel lined with* dead leaves pattern* on the floor (*patterned boxes*, data from Linander et al. 2016) or of bees flying over a white floor (*white boxes*). *Boxes* indicate the extent of the 25–75% interquartile range, the *horizontal line* indicates the median, *whiskers* indicate the full extent of the data, and *red crosses* represent outliers. *Asterisks* indicate the significance level (Kruskal–Wallis test): **p < 0.01, ***p < 0.001

We have previously suggested that, when the distance to nearby lateral surfaces is large, bumblebees use ventral optic flow cues to control ground speed (Linander et al. 2016). To investigate the role of ventral optic flow cues on the control of lateral position in tunnels of different width, we compared the flight trajectories of bumblebees flying over a floor generating strong optic flow cues (dead leaves pattern, see Fig. 1c, note that data from this condition are re-analysed and re-plotted from Linander et al. 2016) with trajectories of bumblebees flying over a floor generating only minimal optic flow cues (white floor, see Fig. 1a). When both walls were lined with the dead leaves pattern, the lateral positions remained relatively close to the midline in both the absence and presence of ventral optic flow (Table 1). However, in comparison to when the floor provided strong optic flow cues, the lateral positions were generally more dispersed across the tunnel width and were less concentrated in the central quarter of the tunnel when the floor was white [five central bars in Fig. 4a: 64 vs. 88% (60 cm); 71 vs. 70% (120 cm); 53 vs. 67% (180 cm) and 44 vs. 71% (240 cm)]. The within-flight variation in lateral position was also significantly larger when the floor was white than when it displayed the dead leaves pattern (Fig. 4b, Kruskal–Wallis *p* < 0.001 (60–180 cm) and *p* < 0.01 (240 cm); for statistical details, see Table 1), suggesting that the flight paths become wider when ventral optic flow cues are minimized. This is supported by the result showing that the flight paths are significantly more torturous for bees flying over a white floor than for bees flying over a patterned floor (Wilcoxon rank-sum *p* < 0.001; for statistical details, see Table 2). Together, these results show that ventral optic flow improves position control and helps bumblebees to maintain straighter flight trajectories.

**Table 2:** Details of tortuosity and statistical analyses

Tunnel width (cm)	Condition			Tortuosity median (interquartile range)	Wilcoxon statistics (a vs. b; **b vs. c; *a vs. c)
	Floor		Walls		
60	a	White	Dead leaves	1.036 (1.020–1.052)	*Z* = 8.2, *p* < 0.001
	b	Dead leaves	Dead leaves	1.010 (1.006–1.018)	
120	a	White	Dead leaves	1.020 (1.011–1.036)	*Z* = −5.8, *p* < 0.001
	b	Dead leaves	Dead leaves	1.008 (1.004–1.016)	
180	a	White	Dead leaves	1.024 (1.013–1.052)	*Z* = 7.7, *p* < 0.001
	b	Dead leaves	Dead leaves	1.006 (1.002–1.014)	Z** = 4.7, *p* < 0.001
	c	Dead leaves	Grey	1.014 (1.006–1.024)	Z* = −4.4, *p* < 0.001
240	a	White	Dead leaves	1.022 (1.008–1.043)	*Z* = 3.3, *p* < 0.001
	b	Dead leaves	Dead leaves	1.013 (1.005–1.027)	

Data from the condition with a patterned floor and patterned walls (b) are re-analysed from Linander et al. (2016)

* White floor and patterned walls vs. patterned floor and grey walls (a vs. c)

** Patterned floor and patterned walls vs. patterned floor and grey walls (b vs. c)

**Fig. 5:**
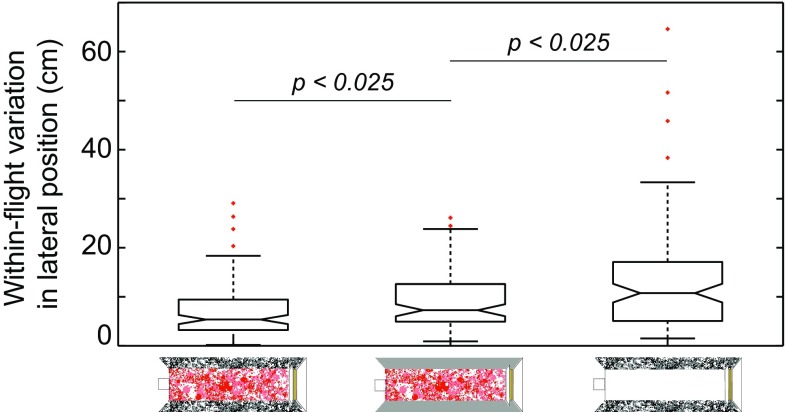
Effect of removing translational optic flow cues in the *lateral* or *ventral* visual fields. The tunnel was 180 cm-wide lined with the *dead leaves pattern* on the walls and floor (*left*), *dead leaves pattern* on the floor and *grey* walls (*middle*), or *dead leaves pattern* on the walls and *white* floor (*right*) (see also the tunnel sketches below each *boxplot*). The condition with a patterned floor and patterned walls (*left*) is data re-analysed from Linander et al. (2016). The plots show the within-flight variation (25–75% interquartile range) in lateral position for each condition. *Boxes* indicate the extent of the 25–75% interquartile range, the *horizontal line* indicates the median, *whiskers* indicate the full extent of the data and *red crosses* represent outliers

To investigate if bumblebees use ventral optic flow cues alone when the distance to nearby lateral surfaces are large, we compared trajectories of bees flying in a 180 cm-wide tunnel generating strong lateral and ventral optic flow cues (patterned floor and walls, see Fig. 1c) with flights in a 180 cm-wide tunnel generating only ventral optic flow cues (patterned floor and uniformly grey walls, see Fig. 1d). If bees are controlling their position using ventral optic flow cues alone, we would not expect to see any significant difference in the within-flight variation in lateral position or tortuosity of the flight paths. In the absence of lateral optic flow cues, the within-flight variation was slightly larger (Fig. 5a, Kruskal–Wallis $\chi_{1/206}^{2}$ = 10.6, *p* = 0.0012) and the flight paths more torturous (Wilcoxon rank-sum *p* < 0.001; for statistical details, see Table 2) than when these cues were present. This suggests that lateral position control becomes worse in the absence of lateral optic flow cues. To further investigate in which field of view that the translational optic flow has greatest impact, we compared flights down the tunnel when it generated only lateral optic flow cues (white floor and patterned walls, see Fig. 1a) with flights down the tunnel when it generated only ventral optic flow cues (see Fig. 1d). Interestingly, the within-flight variation in lateral position was significantly smaller when only ventral optic flow cues were present, compared to when only lateral optic flow cues were available (Fig. 5a, Kruskal–Wallis $\chi_{1/193}^{2}$ = 5.3, *p* = 0.021). The flight paths were also significantly straighter (less tortuous) when only ventral optic flow cues were present (Wilcoxon rank-sum *p* < 0.001; for statistical details, see Table 2). The four test results reported above have a Bonferroni corrected significance level of *p* < 0.025. These results indicate that, while the control of lateral position is slightly impaired in the absence of lateral optic flow cues in wider tunnels, bumblebees are nonetheless much better at controlling their lateral position and maintaining a straight flight path in the presence of ventral rather than lateral optic flow cues.

Given that ventral optic flow cues alone provide sufficient information for position control, do bumblebees prefer flying over textured ground rather than over featureless surfaces? To investigate this, we presented the dead leaves pattern on only one half of the 180 cm-wide tunnel, such that only one side of the floor (right or left) provided ventral optic flow cues, while the other half was white (see Fig. 1e, f). Both walls were uniformly grey to minimize lateral optic flow cues. Irrespective of the side that the pattern was presented on, the bees shifted their lateral position so as to fly over it [pattern on the left side: 7.5 (−5.0 to 20.3) cm; pattern on the right side: −6.9 (−16.0 to 9.9) cm; median (interquartile range); Wilcoxon rank-sum *Z* = 3.4, *p* < 0.001). This suggests that bumblebees do adjust their position so as to maintain optic flow cues in their ventral visual field.

## Discussion

### Centring performance in environments of different proximity

When flying down the two narrower tunnels (60 and 120 cm-wide), the bees positioned themselves along the midline of the tunnel at an equal distance to each wall (Fig. 2a, b; Table 1). When the lateral optic flow cues on one side were minimized using a grey pattern, the bees adjusted their position to fly closer to the grey wall (Fig. 3; Table 1). Because the patterned wall generates a higher magnitude of optic flow than the grey wall, bumblebees increased their distance from it in an apparent attempt to equalise the magnitude of optic flow experienced in each eye. This result is consistent with the “centring response” hypothesis, first described in experiments performed on honeybees flying in narrow tunnels (Kirchner and Srinivasan 1989; Srinivasan et al. 1991, 1996), suggesting that position is controlled by balancing the magnitude of optic flow experienced in the left and right lateral visual fields. Our results are also consistent with studies performed on bumblebees flying in narrow tunnels (Dyhr and Higgins 2010; Baird et al. 2011; Linander et al. 2015) and flies (Kern et al. 2012) as well as with a similar study performed on birds (Bhagavatula et al. 2011). Interestingly, the importance of lateral optic flow cues for position control decreased as tunnel width increased to 180 and 240 cm. First, the within-flight variation in lateral position increased, and the flight trajectories became less centred and were instead more dispersed across the tunnel width (Fig. 2). Second, the removal of optic flow cues from one wall had no significant effect on lateral position in these wider tunnels (Fig. 3; Table 1), suggesting that the bees were no longer balancing the lateral optic flow cues. It is not possible from our data to determine exactly at what distance from the two walls that the bees cease to balance the magnitude of optic flow in each visual field to keep an equal distance to the walls, but it appears to lie somewhere between 120 and 180 cm.

Serres et al. (2008) found that, when flying in a 95 cm-wide tunnel, honeybees will adopt a wall-following behaviour rather than a centring behaviour if the entrance and the feeder are positioned close to one of the walls. Based on these results, the authors suggest that flight position is controlled by a ‘unilateral optic flow regulator’ that will generate a wall-following behaviour when the distances to nearby lateral surfaces are large. Interestingly, we found no evidence for such wall-following behaviour in our study. Even in the widest tunnel (more than 2.5 times as wide as in the study by Serres et al. 2008), the lateral positions were dispersed along the width of the tunnel, rather than being concentrated along one of the walls. Further studies are needed to understand if these disparate observations are due to differences in experimental design or inter-species differences.

### The importance of ventral optic flow for position control

Earlier studies have demonstrated the importance of ventral optic flow for flight speed and height control in bees (Srinivasan et al. 1996, 2000; Baird et al. 2006; Barron and Srinivasan 2006; Portelli et al. 2010, 2011; Linander et al. 2016). Our present results broaden the understanding of optic flow-based flight control by showing that, when the distance to nearby lateral surfaces is large, ventral optic flow can instead be used to prevent large lateral deviations and to maintain a straight course. We found that, in the presence of ventral optic flow cues, bumblebee flight trajectories were more densely centred around the midline and the within-flight variation in lateral position was smaller compared when ventral optic flow was absent (Fig. 4). The flight paths were also straighter in the presence of strong ventral optic flow cues (Table 2). It is possible that the increased tortuosity over a white floor is a result of the bees increasing their lateral movements in an attempt to use lateral optic flow from the walls. If this was the case, then we would expect the flight paths to be centred about the midline with large lateral deviations to each side. However, the shape and position of the individual flight paths (Fig. 2a) do not support this explanation. Thus, the increased tortuosity in wider tunnels with a white floor is more likely an effect of the reduced optic flow information available in the ventral visual field, which limits the bees from being able to detect and control lateral deviations in their flight path. Ventral optic flow thus seems to be an important cue for position control, especially in open environments.

When lateral optic flow cues were minimized, the bumblebees still safely navigated down the tunnel to the food reward, demonstrating that they can control their flight using ventral optic flow cues alone. However, the flight trajectories were straighter and the within-flight variation in lateral position was slightly smaller in the presence of both lateral and ventral optic flow cues (Table 2; Fig. 5), indicating that when lateral optic flow cues are available, they are still used (in combination with ventral optic flow) to minimize the sideways deviations of the flight path. The finding that bumblebees were much worse in controlling lateral position in a 180 cm-wide tunnel generating only lateral optic flow cues, than in a tunnel of similar width generating only ventral optic flow cues (Table 2; Fig. 5), suggests that the ventral optic flow field is the most reliable cue for position control in wider environments.

From the ventral optic flow field, insects can get information about their forwards, backwards, and lateral or rotational movements over the ground. When flying on a perfectly straight course, a bumblebee will experience a ventral optic flow field moving exclusively in the front-to-back direction. However, as soon as the bee is translating slightly towards the right side or the left side, the ventral optic flow field vectors will gain a leftwards or rightwards component. In combination with the well-studied optomotor response (Götz 1975; Reichardt and Poggio 1976), that compensates for unintended deviations off course by generating a turning response with the same direction and magnitude as the rotational optic flow field, changes in the translational component of the ventral optic flow field can also provide the bee with information to correct for unwanted deviations in their flight course. In the bees’ natural environment, the ability to keep a straight course towards a food source (or a nest), well away from obstacles, would result in faster, more efficient, and safer foraging flights.

In most terrestrial environments, the ground is heavily textured and can provide insects with clearly perceivable ventral optic flow information. The only time when this might change is when flying over a flat texture-less surface such as a pond or a lake. Honeybees trained to fly over still water tend to fly so low that they eventually crash into the water surface (Heran and Lindauer 1963), indicating that they do not cope well in environments poor in ventral optic flow. Furthermore, honeybees are generally hesitant to fly over water and will most likely choose a detour over land if possible (Pahl et al. 2011). Our finding that bumblebees prefer to fly over surfaces that generate strong ventral optic flow cues, rather than over a texture-less ground, is consistent with these observations and suggest that the honeybees’ preference to fly over land may, in part, be due to their reliance on ventral optic flow cues to control their flight in open environments.

While the availability of optic flow cues in the lateral visual field might be very variable depending on the distance to nearby lateral surfaces, the availability of ventral optic flow cues depends only on the height at which the insect flies. Our results suggest that, when the distance to nearby lateral surfaces is small (such as in narrow corridors), bumblebees control their position by balancing the magnitude of lateral optic flow experienced in each eye. When the distance to nearby lateral surfaces is large (such as in wider corridors), however, bumblebees rely on information embedded in the ventral optic flow field, which they can use effectively to maintain a straight flight path. In this way, bumblebees can flexibly adapt, where in the visual field, they use optic flow for flight control depending on the proximity of nearby surfaces, enabling them to navigate safely in a broad range of environments.
